# A Comparative Analysis of Short‐Term Outcomes of Off Pump Coronary Artery Bypass Grafting Alone or Simultaneous Cerebral Revascularization: A Single‐Center Retrospective Study

**DOI:** 10.1002/clc.70373

**Published:** 2026-06-11

**Authors:** Bo Li, Qing‐Rui Fang, Bo‐Chen Yao, Qing‐Liang Chen, Jun‐Shan Li, Qiang Wang, Feng Zhao, Dong Wei, Dong‐Yan Yang, Jing Sun, Zhi‐gang Guo

**Affiliations:** ^1^ ICU Department of Cardiovascular Surgery Tianjin University Chest Hospital Tianjin China; ^2^ Department of Cardiovascular Surgery Tianjin University Chest Hospital Tianjin China

**Keywords:** carotid artery stenting, carotid endarterectomy, combined cardio‐cerebrovascular intervention, off pump coronary artery bypass grafting, short‐term prognosis

## Abstract

**Objectives:**

The impact of simultaneous coronary artery bypass grafting (CABG) with cerebral revascularization versus CABG alone on perioperative complications and short‐term outcomes remains controversial in patients with simultaneous coronary and cerebrovascular disease. Our study was specifically designed to evaluate the effects of simultaneous cardio‐cerebrovascular revascularization versus isolated off‐pump CABG (OPCABG) in patients with severe carotid stenosis.

**Methods:**

This retrospective analysis included patients undergoing OPCABG between May 2023 and May 2025. Participants were divided into two groups based on simultaneous carotid revascularization status, including the combined cardio‐cerebrovascular revascularization group (*n* = 57) and the isolated OPCABG control group (*n* = 60). The primary endpoints comprised 30‐day mortality and all‐cause mortality during follow‐up. Cardiac, hepatic, and renal function biomarkers were monitored daily for three consecutive postoperative days. Kaplan−Meier analysis demonstrated the survival probability of patients undergoing different surgical procedures throughout the follow‐up period.

**Results:**

Our study with a median follow‐up of 11.5 months (IQR: 6.8−20.0 months) observed a lower short‐term mortality rate of 5.3% (3/57) in the combined cardio‐cerebrovascular revascularization group. Our results demonstrated no significant differences in 30‐day mortality and all‐cause mortality during follow‐up between the combined cardio‐cerebrovascular intervention and isolated OPCABG. No intergroup differences were observed in cardiac, hepatic, or renal function within the first three postoperative days. The combined surgical approach reduced hospitalization duration.

**Conclusion:**

Our findings demonstrated comparable short‐term outcomes between patients receiving simultaneous cardio‐cerebrovascular revascularization and those undergoing isolated CABG. This simultaneous intervention represents a clinically meaningful advancement in managing systemic vasculopathy.

## Graphical abstract

The final flowchart categorizes subjects into two groups: the control group (*n* = 60) undergoing OPCABG alone and the combined cardiocerebrovascular revascularization group (*n* = 57).

## 1 Introduction

Cardiovascular and cerebrovascular diseases pose a critical public health threat to the Chinese population, claiming approximately 4 million deaths annually and accounting for over 40% of total deaths, with stroke and ischemic heart disease (IHD) representing the primary diseases [1, 2, 3]. Notably, patients undergoing coronary artery bypass grafting (CABG) with 6% to 14% present with severe carotid disease [4, 5]. Perioperative stroke, among the most devastating complications of CABG with a mortality rate reaching 24.8%, is independently associated with significant carotid stenosis [4]. Consequently, various treatment strategies, including staged or simultaneous carotid revascularization via carotid endarterectomy (CEA) or carotid artery stenting (CAS), have been proposed for patients with simultaneous pathologies [4, 6, 7, 8, 9]. However, no consensus exists regarding the optimal approach or comparative prognostic outcomes of these strategies.

Carotid revascularization combined with CABG has been widely used to reduce the risk of stroke and death [4, 10]. Evidence from some studies points to no significant difference in mortality, stroke, myocardial infarction (MI), postoperative cognitive dysfunction (POCD) or major adverse cardiovascular events (MACEs) between simultaneous CABG/CEA and staged procedures during 30‐day and extended follow‐up [7, 8, 11, 12]. However, there is also evidence indicating that this staged surgical approach may increase the risk of postoperative cardiac complications [13]. Emerging evidence also suggests reduced cerebral activity levels in patients undergoing simultaneous CABG and CEA [7]. A meta‐analysis demonstrated significantly lower 30‐day mortality and perioperative stroke rates with staged CAS and CABG compared to synchronous procedures [14].

This retrospective analysis examined 57 patients with severe cerebrovascular stenosis undergoing combined procedures and 60 patients receiving isolated off‐pump CABG (OPCABG). Our objective was to compare the short‐term clinical benefits of combined cardio‐cerebrovascular intervention versus isolated OPCABG.

## 2 Materials and Methods

### 2.1 Study Design and Participants

**Figure 1:**
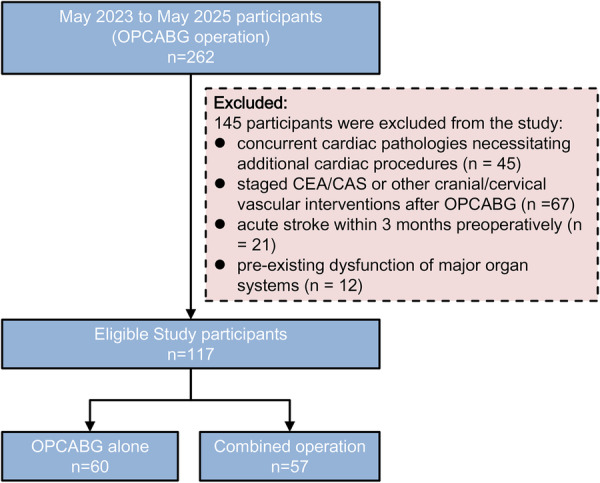
Flowchart of subject inclusion. CAS, carotid artery stenting; CEA, carotid endarterectomy; OPCABG, off‐pump coronary artery bypass grafting.

This retrospective consecutive cohort study analyzed 117 patients who underwent OPCABG operation at the Tianjin University Chest Hospital from May 2023 to May 2025. The average follow‐up time was 11.5 months (IQR: 6.8−20.0 months). This study was approved by the Ethics Committee of Tianjin University Chest Hospital (No. 2025LW‐27) (Tianjin, China). Participants were stratified into two cohorts, including control group (*n* = 60) with severe cerebrovascular stenosis or occlusion patients who did not receive simultaneous neurosurgical intervention and combined cardio‐cerebrovascular revascularization group (*n* = 57) with patients undergoing simultaneous procedures for severe cerebrovascular stenosis/occlusion via CAS + OPCABG (*n* = 22), CEA + OPCABG (*n* = 33), or superficial temporal artery to middle cerebral artery (STA‐MCA) bypass + OPCABG (*n* = 2) (Figure 1). Inclusion criteria [1]: the subjects received OPCABG operation [2]; preoperative confirmation of unilateral/bilateral carotid artery stenosis (70%−99%), occlusion, or other severe cerebrovascular stenoses via cervical vascular ultrasound and computed tomography angiography (CTA). Exclusion criteria [1]: simultaneous cardiac pathologies necessitating additional cardiac procedures [2]; complex cerebrovascular disease requiring staged CEA/CAS or other cranial/cervical vascular interventions after OPCABG [3]; acute stroke within 3 months preoperatively [4]; pre‐existing dysfunction of major organ systems (pulmonary, hepatic, or renal). CAS is primarily indicated for surgically high‐risk patients with anatomically favorable lesions (e.g., stenosis at C1−C2 segment, non‐circumferential calcification, normal aortic arch morphology), particularly symptomatic individuals (TIA/stroke) aged ≤ 70 years; it should be avoided in patients with type III/bovine aortic arch, ulcerated plaques, or simultaneous atrial fibrillation. CEA, as the gold standard, is preferred for severe bifurcation stenosis (symptomatic ≥ 70% or asymptomatic ≥ 80%) with unstable plaque characteristics (ulceration/thrombus), especially in patients ≥ 70 years who derive greater long‐term benefit; contraindications include prior radical neck radiation and severe cardiopulmonary comorbidities precluding general anesthesia. STA‐MCA bypass is reserved for strictly selected cases of hemodynamic failure due to intracranial chronic arterial occlusion, requiring significantly impaired cerebrovascular reserve, elevated oxygen extraction fraction, and recurrent ischemic events refractory to medical therapy. Typical candidates include advanced Moyamoya disease or symptomatic MCA occlusion; it is contraindicated if the recipient vessel diameter is <0.8 mm or in extensive cerebral infarction.

### 2.2 Primary and Secondary Endpoints

The primary endpoint of this study was all‐cause mortality, encompassing both 30‐day mortality and all‐cause mortality during the postoperative follow‐up period. Secondary endpoints included the incidence of in‐hospital stroke, MI, postoperative incidence of transient ischemic attack, angina pectoris, and duration of hospitalization.

### 2.3 Surgical Operation

In our cohort population, all eligible patients underwent either synchronous carotid revascularization (CEA/CAS) with OPCABG or combined STA‐MCA bypass and OPCABG, wherein CEA or CAS procedures were completed before initiating OPCABG, while STA‐MCA bypass was systematically performed after OPCABG. Patients presenting with acute MI received definitive surgical intervention following a mandatory 4‐week post‐infarction recovery period.

For CEA procedures, patients were positioned supine with the head rotated contralateral to the surgical site. Following sterile preparation and draping, an oblique incision was meticulously marked along the anterior border of the sternocleidomastoid muscle on the operative side. The incision was carried through the skin and platysma, with subsequent layered dissection and retraction to expose the carotid sheath under microsurgical visualization. The sheath was incised to mobilize the common carotid artery, internal carotid artery, external carotid artery, and superior thyroid artery. Temporary occlusion clamps were sequentially applied to these vessels. An arteriotomy was performed longitudinally, enabling systematic plaque removal with continuous irrigation using heparinized saline solution to evacuate atheromatous debris. Arterial closure was achieved via precision suturing under direct visualization to confirm hemostasis and vessel patency. Wound closure proceeded in anatomical layers from deep fascial planes to cutaneous sutures.

During CAS procedures, patients were maintained under general anesthesia with endotracheal intubation while an 8 F arterial sheath was inserted into the right common femoral artery. Diagnostic cerebral angiography was performed to assess stenosis location and severity. Following systemic heparinization, an arterial stent was deployed based on angiographic findings. Completion angiography confirmed residual stenosis < 30% with restoration of vascular luminal patency. The percutaneous access site was sealed using a vascular closure device.

For STA‐MCA bypass procedures, microsurgical dissection commenced with scalp incision followed by isolation of the main trunk and frontal branch of the right STA. Minor tributaries were cauterized and divided, with the distal STA segment transected, luminal irrigation performed using heparinized solution, and the vessel temporarily clamped for graft preparation. Subsequently, the temporalis muscle was incised and retracted. A craniotomy was performed with dural tack‐up sutures placed circumferentially; the dura was opened in a curvilinear fashion to allow microsurgical dissection of the Sylvian fissure. Upon exposure of the middle cerebral artery (MCA), temporary clips were applied proximal and distal to the target site. An arteriotomy was created longitudinally, and luminal irrigation with heparinized papaverine solution was conducted. End‐to‐side anastomosis between the STA graft and MCA was completed, confirming watertight closure without bleeding. Intraoperative angiography demonstrated patency of the bypass graft. Closure included dural suturing, titanium miniplate fixation of the bone flap, and layered reapproximation of the temporalis muscle, subcutaneous tissue, and skin.

For OPCABG procedures, a median sternotomy was performed via a midline incision. The left internal mammary artery (LIMA) was routinely anastomosed to the left anterior descending (LAD) coronary artery. Conduit harvesting included endoscopic or open harvest of the great saphenous vein from the lower limb for anastomosis to additional target vessels. After confirming patency of all graft anastomoses and absence of active bleeding, layered closure of the chest was achieved with sternal fixation using sternal wires, followed by fascial and cutaneous suturing. All procedures consistently employed a no‐clamp technique for proximal anastomosis: Following median sternotomy, the pericardium was retracted laterally using the ThoraTrak sternal retractor system to expose the anterior ascending aorta. The Enclose II proximal anastomotic device (Model AN‐1401; Medtronic, USA) created a 4.0−4.5 mm aortotomy without partial clamping to minimize atherosclerotic plaque disruption. Continuous suturing with 7‐0 polypropylene (Prolene) was performed under bloodless visualization maintained by a CO_2_ blower at 8–10 L/min flow. For target vessel stabilization, the Octopus IV tissue stabilizer (Medtronic) with negative‐pressure suction (−300 to −400 mmHg) was applied. Cardiac positioning was optimized through synergistic adjustment of deep pericardial traction sutures and rightward table tilting (15°−20°), ensuring complete exposure of target territories—particularly the obtuse marginal branches. Cerebral protection protocols included: Continuous warm CO_2_ insufflation (37°C at 5 L/min) into the surgical field; strict maintenance of mean arterial pressure ≥ 70 mmHg; and immediate deployment of the HemoStent proximal shunt when regional cerebral oxygen saturation (rSO_2_) decreased > 20% from baseline.

### 2.4 Anticoagulant Therapy

For all patients scheduled for combined CAS and OPCABG, dual antiplatelet therapy (DAPT) comprising aspirin 100 mg and clopidogrel 75 mg daily is initiated 1 week preoperatively. Postoperatively in the ICU, anticoagulation is switched to subcutaneous low‐molecular‐weight heparin alongside aspirin 100 mg antiplatelet therapy; upon transfer to the general ward, DAPT (aspirin + clopidogrel) is resumed and continued for 1 year post‐discharge before transitioning to aspirin monotherapy based on individualized risk assessment. Patients undergoing CEA with OPCABG receive no preoperative DAPT but follow identical postoperative anticoagulation and antiplatelet regimens as CAS patients. For those scheduled for STA‐MCA bypass with OPCABG, aspirin 100 mg daily is administered from admission until the day preceding surgery, with postoperative management mirroring the CAS protocol regarding anticoagulation and antiplatelet strategies.

### 2.5 Clinical Data Collection and Measurements

The variables considered in this study included patient demographics, such as age and gender, New York Heart Association (NYHA) classification, disease history, carotid artery lesion, and ultrasound measurement parameters. Clinical data were collected from the electronic medical record system. All specimens were tested within 2 h following their collection. In cases for which immediate testing was not feasible, specimens were stored at a temperature of –20°C for a duration not exceeding 2 days. Cardiac troponin T (cTnT), total bilirubin (TBIL), creatinine (Cr), alanine aminotransferase (ALT), and aspartate aminotransferase (AST) were conducted in the cobas e 801 automatic immunoassay analyzer (Roche, Basel, Switzerland) along with original reagents. All aforementioned measurements were performed in accordance with the manufacturer's instructions.

### 2.6 Statistical Analysis

A database was established using Epidata 3.1 for data entry, employing a double data entry method, followed by verification, organization, and saving. Before conducting formal statistical analysis, a comprehensive cleaning of all collected data was performed. Data integrity was checked to ensure there were no missing values, outliers, or incorrectly entered data. The missing rate was calculated for each variable, and parameters with missing values exceeding 30% (threshold for secondary variables) or 15% (threshold for key predictors) were excluded. The missing mechanism was diagnosed using Little's MCAR test combined with clinical judgment to distinguish between types of missingness: For MCAR (Missing Completely at Random) variables, continuous variables were imputed with the median, and categorical variables with the mode. For MAR (Missing at Random) variables, multiple imputation by chained equations (MICE, with *m* = 5 iterations) was applied. For MNAR (Missing Not at Random) variables, a sensitivity analysis was conducted using pattern mixture models. Outlier handling: continuous variables were winsorized using Tukey's rule (Q1 to 1.5 × IQR to Q3 + 1.5 × IQR), and categorical variables were checked for logical errors. Normality distribution was tested using the Kolmogorov–Smirnov test, and homogeneity of variance was assessed using Levene's test. Measurement data that conformed to a normal distribution were expressed as the mean ± standard deviation, and intergroup comparisons were conducted via *t* test. Categorical data are expressed as frequencies with constituent percentages. Group comparisons were performed using chi‐square (*χ* ^2^) test or Fisher's exact test. Patient survival rates were calculated via the Kaplan−Meier method, with inter‐group differences assessed by log‐rank testing. For prognostic factor analysis, variables demonstrating potential significance (*p* < 0.1) in univariate Cox proportional hazards models were incorporated into multivariate Cox regression analyses. Similarly, preoperative factors with *p* < 0.1 were subjected to multiple linear regression modeling to evaluate their relationship with total hospital length of stay. Statistical significance was defined as a two‐sided *p* value < 0.05.

## 3 Results

### 3.1 Baseline Characteristics of the Cohort Population

Patients were stratified based on OPCABG postoperative cerebrovascular revascularization status into a simultaneous cardio‐cerebrovascular intervention cohort (also known as combined treatment group; *n* = 57) and an isolated OPCABG control group (*n* = 60). The combined treatment group averaged 68.2 ± 6.9 years with 45 males (78.9%), while controls averaged 71.2 ± 4.7 years with 47 males (78.3%). Comparative analysis demonstrated no statistically significant differences in baseline characteristics, including sex, smoking history, NYHA classification, hypertension, diabetes mellitus, stroke history, MI history, prior PCI, atrial fibrillation, triple‐vessel disease, carotid occlusion, left atrial end‐diastolic diameter (LAEDD), left ventricular end‐diastolic dimension (LVEDD), or LVEF. Significant intergroup disparities were observed in bilateral carotid stenosis prevalence (*p* = 0.005) and mean age (*p* = 0.007) (Table 1).

**Table 1:** Baseline characteristics of the cohort population.

Variables	Combined group (*n* = 57)	OPCABG (*n* = 60)	*t*/*χ* ^2^	*p*
Male (*n*, %)	45 (78.9%)	47 (78.3%)	0.01	0.94
Age (years)	68.2 ± 6.9	71.2 ± 4.7	−2.77	0.007
Smoking history (*n*, %)	25 (43.9%)	21(35.0%)	0.96	0.33
*NYHA classification (n, %)*				
Ⅰ	11 (19.3%)	13 (21.7%)	0.10	0.75
Ⅱ	43 (75.4%)	45 (75.0%)	0.00	0.96
Ⅲ	3 (5.3%)	2 (3.3%)	0.00	0.95
*Diseases history (n, %)*				
Hypertension	40 (70.2%)	48 (80.0%)	1.51	0.29
Diabetes mellitus	34 (59.6%)	32 (53.3%)	0.47	0.49
Stroke	17 (29.8%)	14 (23.3%)	0.63	0.43
Myocardial infarction	12 (21.1%)	21 (35.0%)	2.81	0.09
PCI	12 (21.1%)	7 (11.7%)	1.89	0.17
Atrial fibrillation	3 (5.3%)	1 (1.7%)	1.15	0.29
Triple vessel disease	38 (66.7%)	49 (81.7%)	3.45	0.06
*Carotid artery lesion (n, %)*				
occlusion	12 (21.1%)	19 (31.7%)	1.69	0.17
Bilateral carotid artery stenosis	16 (28.1%)	5 (8.3%)	7.73	0.005
*Ultrasound measurement*				
LAEDD (mm)	38.7 ± 6.9	38.8 ± 4.7	−0.04	0.97
LVEDD (mm)	51.8 ± 4.8	52.0 ± 5.4	−0.28	0.78
LVEF (%)	56.3 ± 6.9	55.5 ± 8.3	0.56	0.57

Abbreviations: LAEDD, left atrial end‐diastolic diameter; LVEDD, left ventricular end‐diastolic dimension; LVEF, left ventricular ejection fraction; NYHA, New York Heart Association; OPCABG, off‐pump coronary artery bypass grafting; PCI, percutaneous coronary intervention.

### 3.2 Postoperative Evolution of Cardiac, Renal, and Hepatic Function Parameters

**Figure 2:**
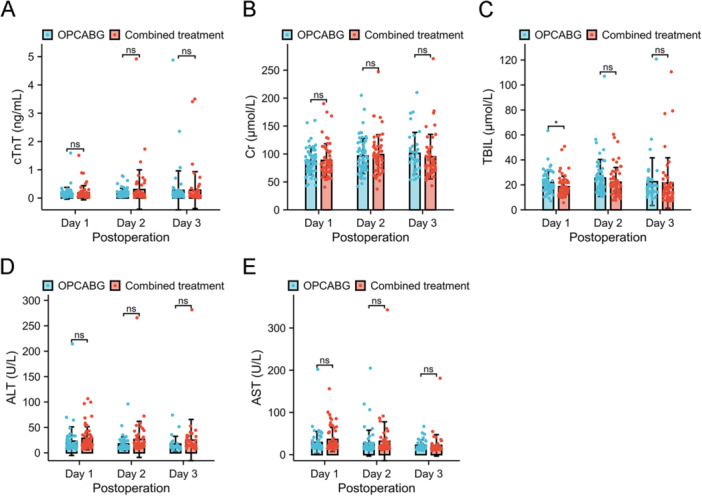
Postoperative evolution of cardiac, renal, and hepatic function parameters. Serum levels of cTnT (A), Cr (B), TBIL (C), ALT (D), and AST (E) were measured daily on postoperative Days 1, 2, and 3 in both the simultaneous cardio‐cerebrovascular revascularization cohort and the isolated OPCABG control group. *p < 0.05. ALT, alanine aminotransferase; AST, aspartate aminotransferase; Cr, creatinine; cTnT, cardiac troponin T; ns, no significant; OPCABG, off pump coronary artery bypass grafting; TBIL, total bilirubin.

Both cohorts exhibited mildly elevated cTnT levels on postoperative day 1, with concentrations of 0.19 ± 0.25 ng/mL in the combined treatment group versus 0.17 ± 0.21 ng/mL in the isolated OPCABG control group. By postoperative Day 2 and 3, no significant differences in cTnT were also observed in the two groups (Figure 2A). Serial assessment demonstrated comparable Cr levels between groups during postoperative Days 1−3, with stable renal profiles observed throughout this period (Figure 2B). Hepatic evaluation employed TBIL (Figure 2C), ALT (Figure 2D), and AST (Figure 2E). While significant intergroup disparity in TBIL emerged on postoperative day 1, other hepatic biomarkers showed no statistically differences. These findings collectively establish that the simultaneous cardio‐cerebrovascular intervention cohort and isolated OPCABG control group exhibited clinically comparable postoperative trajectories in cardiac, renal, and hepatic functional parameters.

### 3.3 Postoperative Clinical Parameters

Pre‐discharge ultrasound measurements revealed no significant differences between the combined cardio‐cerebrovascular intervention group and the OPCABG group regarding LAEDD, LVEDD, and LVEF. The ventilator durations were 23.39 ± 51.57 h in the combined group versus 18.27 ± 21.83 h in the OPCABG group, while ICU stays measured 80.33 ± 145.39 h and 63.42 ± 37.69 h, respectively. These metrics indicate delayed extubation timing and prolonged ICU stays in the combined group, though the differences were statistically non‐significant. Notably, the combined group demonstrated significantly shorter total hospital length of stay as compared to the OPCABG group (Table 2).

**Table 2:** Postoperative clinical parameters and outcomes.

Variables	Combined group (*n* = 57)	OPCABG (*n* = 60)	*t*/*χ* ^2^	*p*
*Ultrasound measurement before discharge*				
LAEDD (mm)	35.60 ± 5.13	35.60 ± 3.88	0.00	1.00
LVEDD (mm)	48.65 ± 4.22	50.00 ± 4.56	−1.63	0.10
LVEF (%)	54.89 ± 6.45	54.25 ± 5.97	0.55	0.58
*In‐hospital parameters*				
Ventilator duration (h)	23.39 ± 51.57	18.27 ± 21.83	0.71	0.48
ICU length of stay (h)	80.33 ± 145.39	63.42 ± 37.69	0.87	0.39
Hospital length of stay (day)	13.84 ± 8.00	17.83 ± 8.20	−2.67	0.01
*Early outcomes (n, %)*				
30‐day mortality	1 (1.8%)	0 (0.0%)	0.51	0.49
In‐hospital stroke	2 (3.5%)	0 (0.0%)	0.56	0.45
*Short‐term follow‐up outcomes (n, %)*				
Death	3 (5.3%)	2 (3.3%)	0.00	0.95
Transient ischemic attack	0 (0.0%)	2 (3.3%)	0.46	0.50
Angina event	1 (1.8%)	4 (6.7%)	0.73	0.39

Abbreviations: ICU, intensive care unit; LAEDD, left atrial end‐diastolic diameter; LVEDD, left ventricular end‐diastolic dimension; LVEF, left ventricular ejection fraction.

### 3.4 Identify Clinically Relevant Factors Associated with Total Hospital Length of Stay

The aforementioned results demonstrated a significant reduction in total hospital length of stay among patients undergoing combined cardio‐cerebrovascular intervention compared to the OPCABG cohort. To investigate potential preoperative factors and treatment‐modality effects on hospitalization duration, five covariates were incorporated into a multiple linear regression model, including intervention group assignment, age, bilateral carotid artery disease status, history of MI, and triple‐vessel coronary disease. OPCABG (*β* = 3.356; 95% CI: 0.170–6.542; *p* = 0.039) and MI (*β* = 4.237; 95% CI: 0.906–7.568; *p* = 0.013) were identified as two independent factors associated with prolonged hospital stay. In contrast, Age (*β* = 0.039; 95% CI: −0.215 to 0.293; *p* = 0.764), Triple vessel disease (*β* = 0.012; 95% CI: −1.138 to 1.162; *p* = 0.847), and Bilateral carotid artery stenosis (*β* = 0.388; 95% CI: −3.600 to 4.376; *p* = 0.847) showed no significant association with longer hospital stay (Table 3).

**Table 3:** Multiple linear regression analysis.

Variables	Unstandardized coefficients		Standardized coefficients *β*	*t*	*p*	95% CI	Collinearity	
	*β*	SE					Tolerance	VIF
Constant	3.478	8.986		0.387	0.699	14.328−21.285		
Operation type	3.356	1.608	0.203	2.087	0.039	0.170−6.542	0.847	1.180
Age	0.039	0.128	0.028	0.301	0.764	−0.215 to 0.293	0.919	1.088
Triple vessel disease	0.012	0.580	0.002	0.020	0.984	−1.138 to 1.162	0.946	1.057
Bilateral carotid artery stenosis	0.388	2.013	0.018	0.193	0.847	−3.600 to 4.376	0.917	1.090
Myocardial infarction	4.237	1.681	0.230	2.520	0.013	0.906−7.568	0.956	1.046

Abbreviations: CI, confidence interval; SE, standard error; VIF, variance inflation factor.

### 3.5 Perioperative Outcomes and Follow‐Up in Patients with Combined Treatment or OPCABG Alone

**Figure 3:**
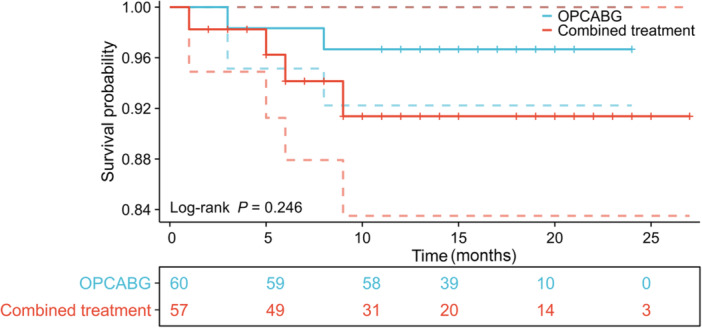
Perioperative follow‐up in patients with the combined cardio‐cerebrovascular revascularization cohort and the isolated OPCABG control group. Kaplan−Meier analysis demonstrates the survival probability of patients undergoing different surgical procedures throughout the follow‐up period. OPCABG, off pump coronary artery bypass grafting.

No statistically significant differences were observed in 30‐day mortality, in‐hospital stroke incidence, or short‐term outcomes, including all‐cause death, transient ischemic attack, and angina events between the two cohorts (Table 2). In the combined cardio‐cerebrovascular intervention group, one patient died of cardiac rupture secondary to postoperative acute MI, while two experienced in‐hospital strokes. All control group patients were discharged successfully. Short‐term follow‐up revealed that both stroke patients in the intervention group died after discharge, with an additional death from lung cancer. The control group had two deaths, one from heart failure due to postoperative MI and one from infection‐induced fever. Transient ischemic attacks occurred in two control patients, and four reported recurrent angina requiring rehospitalization. No transient ischemic attack and only one recurrent angina case were observed in the combined cardio‐cerebrovascular intervention group. Kaplan−Meier analysis showed slightly lower survival probability in the combined cardio‐cerebrovascular intervention group compared to OPCABG alone, though the difference was non‐significant (*p* = 0.246) (Figure 3). Further univariate Cox regression analysis incorporating preoperative variables with *p* < 0.1 demonstrated no significant impact on survival for any of the five covariates (Table 4). Given that the number of events was limited, it is advisable to interpret this result in a guarded manner due to its inherent instability.

**Table 4:** Univariate Cox regression analysis of factors influencing patient prognosis of short‐term mortality.

Variables	*B*	Wald	*p*	HR	95% CI
Operation type	0.971	1.264	0.264	2.64	0.480−14.515
Age	−0.033	0.221	0.638	0.968	0.843−1.110
Bilateral carotid artery stenosis	0.973	1.253	0.263	2.645	0.482−14.520
Myocardial infarction	−3.532	0.856	0.355	0.029	0.000−51.997
Triple vessel disease	−0.126	0.191	0.662	0.882	0.501−1.552

Abbreviations: CI, confidence interval; HR, hazard ratio.

## 4 Discussion

This study primarily compared the prognostic differences between simultaneous revascularization of cerebral and coronary arteries and isolated OPCABG surgery. Our results demonstrated no significant differences in short‐term outcomes between the combined cardio‐cerebrovascular intervention and isolated OPCABG. No intergroup differences were observed in cardiac, hepatic, or renal function within the first three postoperative days. Consequently, we propose that combined management offers potential advantages, including avoidance of perioperative risks from staged surgeries, elimination of secondary anesthetic exposure, reduced need for rehospitalization, and cost‐saving benefits, demonstrating substantial clinical value.

CABG remains a cornerstone intervention for coronary artery disease in cardiac surgery. However, perioperative stroke, as a critical complication with mortality rates approaching 50%, significantly compromises postoperative outcomes. Cerebral vascular stenosis serves as a key risk determinant for such strokes, with incidence escalating to 3%−11% among patients with severe stenosis [5, 10]. This risk exhibits a dose‐dependent relationship with stenosis severity. Contemporary evidence supports prophylactic carotid revascularization preceding CABG and simultaneous cardio‐cerebrovascular revascularization as effective preventive strategies against perioperative stroke [5, 10]. A 10‐year follow‐up study conducted at a single center revealed that 30‐day all‐cause mortality was 6.3%, and postoperative neurologic events occurred with 7.2% transient ischemic attack and 4.5% of disabling stroke [12]. In our study, the combined cardio‐cerebrovascular intervention group demonstrated a 30‐day mortality rate of 1.8% and no transient ischemic attack incidence. These findings indicate that the proposed simultaneous approach yields superior short‐term outcomes compared to the prior study [12]. This observation may be attributed to our exclusive use of OPCABG surgery in all patients [15]. An existing study demonstrated that OPCABG significantly reduces perioperative stroke incidence compared to on‐pump CABG (1.3% vs. 10.3%) [16]. Jia et al. [4] reported a median follow‐up of 6.69 years (IQR: 5.82−7.57 years), revealing a mid‐term mortality rate of 11.4% (10/88) for combined cardio‐cerebrovascular intervention. Their analysis identified NYHA grade IV (HR = 5.01, 95% CI: 1.16−21.64; *p* = 0.03) and previous MI (HR = 5.43, 95% CI: 1.01−29.29; *p* = 0.04) as independent risk factors for mid‐term mortality [4]. In contrast, our study with a shorter median follow‐up of 11.5 months (IQR: 6.8−20.0 months) observed a lower short‐term mortality rate of 5.3% (3/57) and found no association between MI and short‐term mortality. This discrepancy may be attributed to differences in follow‐up duration. Importantly, our data suggest that the combined cardio‐cerebrovascular intervention approach achieves significantly lower all‐cause mortality during the initial postoperative year compared to the historical cohort [4]. Additionally, while isolated OPCABG showed lower mortality (3.3%; 2/60) than combined intervention (5.3%), this difference did not reach statistical significance. Golukhova et al. [8] observed that although 30‐day mortality showed no significant difference between the combined cardio‐cerebrovascular intervention and isolated OPCABG, simultaneous interventions elevated the overall risk of postoperative complications (OR = 2.214, 95% CI: 1.048−4.674; *p* = 0.035) and prolonged mechanical ventilation duration. During their 6‐year follow‐up, however, no significant differences emerged in all‐cause mortality or MACEs between the two strategies [8]. Similarly, our study demonstrated no intergroup differences in 30‐day mortality or all‐cause mortality. Although the combined cardio‐cerebrovascular intervention showed a numerical increase in ventilation time, this difference did not achieve statistical significance compared to isolated OPCABG.

Multiple retrospective studies, randomized controlled trials, and meta‐analyses have collectively indicated that synchronous CEA + CABG procedures correlate with elevated early mortality and stroke risks [17, 18, 19, 20]. Critically, these investigations revealed no significant difference in postoperative stroke or mortality rates between combined CEA + CABG and isolated CABG cohorts, demonstrating that adding CEA to CABG confers no additional benefit for reducing stroke or death in asymptomatic patients [17, 18, 19, 20]. This body of evidence presents conflicting conclusions regarding optimal management. Current clinical consensus therefore reserves prophylactic CEA (performed before or simultaneously with CABG) only for asymptomatic patients meeting strict anatomical criteria, including bilateral severe carotid stenosis (>70%) or unilateral severe stenosis with contralateral carotid artery occlusion [5, 10, 21]. Despite ongoing controversy surrounding surgical approaches for patients with coexisting cardiovascular and cerebrovascular disease, our study exclusively enrolled individuals with severe unilateral or bilateral carotid stenosis (70%−99%), carotid occlusion, or other major cerebral artery stenoses. While the combined cardio‐cerebrovascular intervention demonstrated no significant improvement in clinical outcomes compared to isolated OPCABG, it provided tangible clinical benefits through avoiding repeated anesthetic exposure, eliminating the need for readmission, and enhancing health care cost‐effectiveness without increasing perioperative risks.

Current evidence predominantly addresses carotid artery stenosis, yet cerebrovascular pathology is inherently heterogeneous. Isolated carotid revascularization may inadequately resolve multifocal cerebral arterial disease, potentially explaining the lack of significant perioperative stroke risk reduction in patients undergoing synchronous cardio‐cerebrovascular revascularization [4, 18, 20]. Moreover, the exclusive focus on cervical artery interventions excludes patients with intracranial stenosis, thereby limiting comprehensive stroke prevention strategies for OPCABG candidates. Our advocating “comprehensive cardio‐cerebrovascular revascularization” protocol transcends this gap by including all hemodynamically significant cerebral artery stenosis, providing broader surgical inclusivity and enhanced comparative validity against isolated OPCABG outcomes.

Our study analyzed patients with severe cerebral artery stenosis undergoing either synchronous OPCABG combined with cerebral revascularization or isolated OPCABG. Within 72 h postoperatively, cTnT levels showed no intergroup difference, while renal/hepatic biomarkers demonstrated comparable daily downward trends, indicating absent major perioperative complications despite procedural complexity. Pre‐discharge cardiac function was equivalent, confirming unimpaired cardiac recovery with dual revascularization. Although the combined group exhibited numerically prolonged mechanical ventilation and ICU stays, these aligned with clinical observations of transient neurological symptoms possibly linked to cerebral hyperperfusion syndrome. Crucially, total hospitalization was significantly shorter in the combined cohort, with multivariate regression confirming procedural approach as the primary predictor, demonstrating that simultaneous revascularization accelerates overall recovery.

The shorter hospital stay in the combined treatment group may be associated with the following factors: In experienced centers, simultaneous surgery involves rigorous preoperative planning, including comprehensive assessment of carotid stenosis severity, cardiac function, and surgical risk, along with highly simultaneous postoperative monitoring and rehabilitation protocols. Such coordinated management helps reduce complications, accelerates patient ambulation and functional recovery, and thereby shortens the length of hospital stay [22]. Although the study aimed to compare short‐term outcomes between the two groups, in clinical decision‐making, physicians may tend to select patients with relatively better general condition, fewer comorbidities, and stronger anticipated recovery capacity for the combined surgery. This non‐randomized group allocation bias may have endowed the combined group with inherently greater potential for shorter hospitalization. Even though we emphasized strict patient selection in the manuscript, this factor still warrants consideration when interpreting the results. Under rigorous adherence to the principle of patient autonomy, combined surgical intervention may be pursued after comprehensive shared decision‐making. However, a subset of patients with hemodynamically significant carotid stenosis (≥70%) who remain asymptomatic may electively decline simultaneous revascularization. Carotid revascularization modalities, encompassing CEA and CAS, functionally ameliorate extracranial cerebrovascular obstruction, thereby augmenting cerebral perfusion. Theoretically, this dual approach may mitigate intraoperative cerebral embolic burden during OPCABG through stabilization of carotid plaque hemodynamics. Consequently, reduction in major neurological complications potentially confers downstream benefits in hospitalization duration by averting protracted neurorehabilitation requirements [23, 24]. For asymptomatic carotid stenosis patients scheduled for OPCABG, precise risk stratification is paramount in formulating individualized revascularization strategies. High‐risk cohorts, typically including those with near‐occlusion stenosis (≥80%), rapid stenosis progression, contralateral carotid occlusion, imaging evidence of unstable plaque morphology, or impaired cerebrovascular reserve, may derive benefit from combined revascularization [25]. Intermediate‐risk patients (70%−79% stenosis with stable plaques, robust collateral circulation, and no progression signs) represent a distinct category where optimized medical therapy (intensified statins and antiplatelets) coupled with meticulous intraoperative blood pressure management during urgent OPCABG (e.g., for acute coronary syndrome) may be preferable [26, 27]. Conversely, prohibitive‐risk patients, characterized by advanced age (>80 years), limited life expectancy, or major comorbidities (end‐stage renal disease, severe heart failure), should generally avoid revascularization as peri‐procedural risks likely outweigh long‐term stroke prevention benefits [28]. Crucially, symptomatic carotid stenosis fundamentally alters this paradigm: revascularization (CEA or stenting) is strongly recommended given proven reductions in long‐term stroke risk [29]. Ultimately, irrespective of symptom status, management decisions must emerge from multidisciplinary consensus through holistic risk‐benefit assessment that quantifies individualized stroke risk against OPCABG‐related procedural hazards. In summary, the observed reduction in hospitalization length reflects the potential advantages of simultaneous cardiocerebrovascular revascularization in enhancing therapeutic efficiency and simultaneous perioperative care coordination. However, this finding does not necessarily translate to superior clinical outcomes. These results must be interpreted within the context of rigorous patient selection, execution by experienced surgical teams, and standardized perioperative protocols. Furthermore, the long‐term functional outcomes and survival benefits warrant validation through larger multicenter cohorts with extended follow‐up periods.

There are several limitations of our study. As a single‐center retrospective cohort design, it lacks the methodological rigor inherent in randomized double‐blind trials. Additionally, the modest sample size increases susceptibility to bias. Finally, the median follow‐up duration of 11.5 months (IQR: 6.8−20.0 months) may obscure medium‐ or long‐term outcomes, and extended surveillance periods are warranted in subsequent research. Additionally, the utilization of three distinct surgical approaches in the combined intervention group, namely CEA, CAS, and STA‐MCA bypass, introduces potential intra‐group heterogeneity. In future studies, we plan to address this limitation by expanding the sample size and conducting appropriately stratified analyses to mitigate confounding effects.

## 5 Conclusions

In summary, simultaneous cardio‐cerebrovascular revascularization provides a clinical strategy for patients with simultaneous coronary and cerebral artery disease, significantly reducing total hospitalization duration while demonstrating non‐inferior rates of cardiac/cerebrovascular complications and no increase in postoperative mortality, representing a clinically meaningful advancement in the management of systemic vasculopathy. In this study, cardio‐cerebrovascular combined revascularization encompasses the well‐established CAS/CEA plus OPCABG approach for extracranial carotid disease. This approach broadens the indications for simultaneous cardio‐cerebrovascular intervention, extending from extracranial vascular disease to a subset of patients with strictly selected, drug‐resistant intracranial hemodynamic compromise. Multicenter studies are needed to further validate the long‐term efficacy of intracranial cardio‐cerebrovascular intervention combined with surgery.

## Author Contributions

B.L. and Z.G. designed the study. B.L., Q.F., B.Y., Q.C., J.L., Q.W., F.Z., D.W., D.Y., J.S., and Z.G. performed the literature review, experiments, and statistical analysis. B.L., Q.F., and B.Y. edited the manuscript. All authors have read and approved the final manuscript. B.L. and Z.G. confirm the authenticity of all the raw data.

## Ethics Statement

Our research was approved by the Ethics Committee of Tianjin University Chest Hospital (2025LW‐27).

## Consent

The authors have nothing to report.

## Conflicts of Interest

The authors declare no conflicts of interest.

## Data Availability

The data that support the findings of this study are available from the corresponding author upon reasonable request. The data generated in the present study may be requested from the corresponding author.
